# Variation in Diagnosis, Treatment, and Outcome of Esophageal Cancer in a Regionalized Care System in Ontario, Canada

**DOI:** 10.1001/jamanetworkopen.2021.26090

**Published:** 2021-09-21

**Authors:** Steven Habbous, Olga Yermakhanova, Katharina Forster, Claire M. B. Holloway, Gail Darling

**Affiliations:** 1 Ontario Health (Cancer Care Ontario), Toronto, Canada; 2 Faculty of Medicine, University of Toronto, Toronto, Ontario, Canada; 3 Department of Surgery, Sunnybrook Health Sciences Centre, Toronto, Ontario, Canada; 4 Faculty of Medicine, University of Toronto, Toronto, Ontario, Canada; 5 Division of Thoracic Surgery, Toronto General Hospital, Toronto, Ontario, Canada

## Abstract

**Importance:**

Esophageal cancer remains one of the most deadly cancers, ranking sixth highest among cancers leading to the greatest years of life lost.

**Objective:**

To determine how patients with esophageal cancer are diagnosed and treated in Ontario’s regionalized thoracic surgery centers.

**Design, Setting, and Participants:**

This cohort study included patients diagnosed with esophageal cancer between January 1, 2010, and December 31, 2018, identified from the Ontario Cancer Registry, in a single-payer health care system with regionalization of thoracic surgery in the province of Ontario, Canada.

**Exposures:**

Exposures included incidence of esophageal cancer and stage at diagnosis; time from the first health care visit until treatment; and the use of specialist consultations, endoscopic ultrasonography, positron emission tomography and computed tomography, endomucosal resection, esophagectomy, neoadjuvant therapy, adjuvant therapy, radiation alone, and chemotherapy alone or in combination with other treatment.

**Main Outcomes and Measures:**

Outcome measures included wait times, health care use, treatment, and overall survival. Data were analyzed from March 2020 to February 2021.

**Results:**

There were 10 364 patients (mean [SD] age, 68.3 [11.9] years; 7876 men [76%]) identified during the study period. The incidence of esophageal cancer increased over the study period from 1041 in 2010 to 1309 in 2018, which was driven by a 30% increase in the number of adenocarcinomas. The time from first health care encounter to start of treatment was a median 93 days (interquartile range, 56-159 days). Endoscopic ultrasonography was observed for 12% of patients, and positron emission tomography and computed tomography (CT) in 45%. Use of endoscopic mucosal resection was observed for 8% of patients with stage 0 to I disease. A total of 114 of 547 patients (21%) receiving endoscopic resection had a subsequent esophagectomy. Only 2778 patients (27%) had consultations with a thoracic surgeon, a medical oncologist, and a radiation oncologist, whereas 1514 patients (15%) did not see any of these specialists. Of 3047 patients who had an esophagectomy, those receiving neoadjuvant therapy had better overall survival (median survival, 36 months; 95% CI, 32-39 months) than patients who received esophagectomy alone (median survival, 27 months; 95% CI, 24-30 months) or those who received esophagectomy with adjuvant therapy (median survival, 36 months; 95% CI, 32-44 months) despite significant early mortality (log-rank *P* < .001). There was significant variation in treatment modality across hospitals: esophagectomy ranged from 5% to 39%; esophagectomy after neoadjuvant therapy ranged from 33% to 93%; and esophagectomy followed by adjuvant therapy ranged from 0 to 34% (*P* < .001). Perioperative mortality was higher at 30 days for patients receiving esophagectomy at low-volume centers (odds ratio [OR], 3.66; 95% CI, 2.01-6.66) and medium-volume centers (OR, 2.07; 95% CI, 1.33-3.23) compared with high-volume centers (*P* < .001). A longer wait time until treatment was associated with better overall survival (median overall survival was 15 to 17 days vs 5 to 8 days for patients who received treatment earlier than 30 days vs 30 days or longer after diagnosis; *P* < .001).

**Conclusions and Relevance:**

The results of this cohort study suggest that despite regionalization, there was significant regional variability in volumes at designated centers and in the evaluation and treatment course for patients with esophageal cancer across Ontario.

## Key Points

**Question:**

Was there a uniform approach to diagnosis, staging, and treatment of patients with esophageal cancer after regionalization of thoracic surgery in Ontario, Canada?

**Findings:**

In this cohort study of 10 364 patients, there was significant variation in use of staging tests as well as approach to treatment. Postoperative 30- and 90-day mortality was found to be reduced at high-volume centers.

**Meaning:**

Despite regionalization of thoracic surgery, there remains significant variation between centers in the use of staging tests, treatments, and short-term mortality.

## Teaser

This cohort study assesses variations in the diagnosis, staging, and treatment of patients with esophageal cancer after regionalization of thoracic surgery in Ontario, Canada.

## Introduction

Esophageal cancer remains one of the most deadly cancers, ranking sixth highest among cancers leading to the greatest years of life lost.^1^ In North America, the incidence of squamous cell carcinoma has decreased, but esophageal adenocarcinoma has increased and is now the dominant histology.^2^

Although esophagectomy has been the mainstay of curative intent treatment of esophageal cancer, most patients do not receive esophagectomy either because their disease is too advanced at diagnosis,^3^ they are too frail for surgery,^4,5^ they refuse surgery, or they become too ill during the course of their neoadjuvant treatment to receive subsequent surgery.^6^

For patients with very early stage tumors (stage Tis, T1a without multifocal disease), endoscopic mucosal resection (EMR) is an option.^7,8,9,10^ In patients with locally advanced but nonmetastatic tumors, induction chemotherapy or chemoradiation provides benefit in terms of survival but can also enable complete (R0) resection.^11^ Inaccuracies in clinical staging complicates treatment planning as it may underestimate or overestimate the extent of disease.^12^

To optimize patient outcomes in Ontario, treatment of esophageal cancer is regionalized to thoracic surgery centers that are affiliated with regional cancer centers. Therefore, we expected that there would be relative uniformity regarding how patients with esophageal cancer are diagnosed and treated in the province. Herein, we describe the population-level burden of esophageal cancer in Ontario. Specifically, we estimate the incidence over time, wait times until treatment, treatment patterns, health care use during the diagnostic and pretreatment phases of the cancer care continuum, and regional variation in care and outcomes.

## Methods

### Cohort Ascertainment

Patients with esophageal carcinoma were identified from the Ontario Cancer Registry (OCR) using the *International Classification of Diseases for Oncology, Third Revision* topography code C15 (esophagus) and C160 (gastric cardia). Only patients with malignant neoplasms (behavior code 3) diagnosed between January 1, 2010, and December 31, 2018, were included in this cohort study.

Patients were excluded if they were younger than 18 years or older than 105 years at the time of diagnosis, diagnosed at the time of death or by autopsy, had an invalid Ontario Health card number, or were non-Ontario residents at the time of diagnosis. If a patient had multiple esophageal cancer diagnoses between 2010 and 2018, the first diagnosis within the period was selected.

The overarching goal of this work was to identify gaps in care for quality improvement; therefore, research ethics approval was not required as determined by the Ontario Health (Cancer Care Ontario) privacy office, and patient consent was waived. Our study was reported per the Strengthening the Reporting of Observational Studies in Epidemiology (STROBE) reporting guideline for cohort studies.

### Time Intervals

We defined the diagnostic interval as the time from the earliest health care encounter related to the esophageal cancer workup until diagnosis. The earliest health care encounter was restricted to relevant visits, including diagnostic procedures or consultations within 6 months before diagnosis. Visits with a primary care clinician were not considered. The date of diagnosis was obtained from the OCR, which typically coincides with the date of pathological confirmation of cancer. We defined the pretreatment interval as the time from diagnosis until treatment started, or if no treatment, then 2 months after diagnosis.

### Covariates

Patients were classified as having esophageal adenocarcinoma, squamous cell carcinoma, signet ring cell carcinoma, and other (eTable 1 in the Supplement). Unless otherwise indicated, signet ring cell carcinomas were combined with adenocarcinomas.

Information about diagnosis date, topography, morphology, sex, and patient postal code at the time of diagnosis was obtained from the OCR. Information on race and ethnicity was not collected because it is not recorded in the OCR or any other population-based administrative database in Ontario. Birth date was obtained from the Registered Person Database. Death date information was obtained from the OCR and supplemented with the Registered Person Database.

### Demographic Data and Comorbidities

Neighborhood-level sociodemographic information was obtained from the 2006 census using residential postal code at the time of diagnosis using the Postal Code Conversion File, version 7a (Statistics Canada). Travel times were calculated using the 2016 Census Road Network File from the patient’s postal code at diagnosis and the designated thoracic center. Travel time calculations considered speed limits, 1-way travel, avoidance of tolls, and hierarchy of road type (eg, highways preferred over arterial roads). Charlson comorbidity score was calculated using the information on hospital visits and hospitalizations recorded for each patient in the Canadian Institute for Health Information (CIHI) National Ambulatory Care Database (NACRS) or the CIHI Discharge Abstract Database (DAD) within 3 years of a diagnosis date (excluding cancer and the diagnosis date).

### Staging and Pathology Data

Staging was obtained using the Collaborative Staging database, which relies on staging information provided by hospitals registering the case. We supplemented this staging with the pathological stage ascertained from surgical pathology reports.

To obtain pathological tumor staging (pT) and nodal involvement (pN), cases were linked to the pathology database at Cancer Care Ontario. Any pathology report with a specimen retrieval date within 2 weeks before diagnosis and up to 1 year after the diagnosis date containing at least 1 specimen with topography of C15 or C160 and morphology of interest were captured. If at least 1 surgery pathology report was available (eg, endoscopic resection or esophagectomy), it was used to obtain information on tumor characteristics.

Stage was assigned using the American Joint Committee on Cancer classification rules (7th edition, which was in use at the time the patients were staged).^13^ For cases in which stage was available from the Collaborative Staging database and the pathology reports, the pathological stage was used with 2 exceptions: (1) when Collaborative Staging reported stage IV (stage IV was chosen) or (2) when a patient had neoadjuvant treatment and pathological stage was lower than the collaborative stage.

### Data Sources for Health Care Use and Treatment

We searched the CIHI-DAD (for inpatient procedures), CIHI-NACRS (for outpatient procedures), and the Ontario Health Insurance Program (physician billing) for consultations or visits with various health care professionals and diagnostic tests within 6 months before diagnosis and until first treatment (eTable 2 in the Supplement). Esophagectomy or gastrectomy within 2 weeks before to up to 1 year after diagnosis were identified using procedural codes from the CIHI-DAD or CIHI-NACRS, supplemented with surgical codes from the Ontario Health Insurance Program (eTable 3 in the Supplement). We searched the Activity Level Reporting database for evidence of radiation applied to the chest or abdomen within 1 year after diagnosis. Information on systemic treatment was obtained from multiple data sources, including the Ontario Drug Benefits program database, the New Drug Funding Program database, and the Activity Level Reporting database, restricted to any treatment visit for any antineoplastic therapy. Systemic therapy was also captured using the CIHI-DAD and CIHI-NACRS using the procedure code 1ZZ35 with suffixes HAM (intravenous, intramuscular, subcutaneous, or intradermal approach), CAM (oral approach), or YAM (transdermal or other approach) for antineoplastic agents.

### Statistical Analysis

We present mean (SD), median (interquartile range [IQR]), and proportions, where appropriate. All analyses were conducted using Statistical Analysis Software, version 9.4 (SAS Institute Inc) or ArcGIS, version 10.6 (Environmental Systems Research Institute) at Ontario Health (Cancer Care Ontario). Comparisons between continuous and categorical characteristics between groups were estimated using the Student *t* test and χ^2^ test, respectively. Overall survival was assessed using Kaplan-Meier plots and log-rank *P* values. To estimate the effect of hospital volume on perioperative (30-day and 90-day) mortality we used logistic regression. High-volume centers performed 7 to 19 esophagectomies per year, and low-volume centers performed fewer than 7 esophagectomies per year. Adjusted survival analyses were not conducted because we expected substantive residual confounding by indication for the effect of treatment on survival. In accordance with our privacy policy, all cells with values less than 6 were suppressed. All *P* values were 2-sided, and *P* < .05 was considered significant. Data were analyzed from March 2020 to February 2021.

## Results

After exclusions, 10 364 patients (mean [SD] age, 68.3 [11.9] years; 7876 men [76%]; 2488 women [24%]) were included for analysis (eFigure 1 in the Supplement). A total of 70% of patients had no evidence of existing comorbidity (Table 1). Adenocarcinomas accounted for 68% of all cancers (n = 7059), followed by squamous cell carcinomas (n = 2260; 22%) and signet ring cell carcinoma (n = 429; 4%) (Table 1). The number of cases rose from 1041 in 2010 to 1309 in 2018, which was driven by a 30% increase in the number of adenocarcinomas during the study period (eFigure 2 in the Supplement).

**Table 1.** Participant Sociodemographic and Clinical Characteristics

Characteristic	No. (%)
No.	10 364
Age, mean (SD), y	68.3 (11.9)
Sex	
Male	7876 (76)
Female	2488 (24)
Rurality^a^	
Urban	8680 (84)
Rural	1684 (16)
Income quintile^a^	
Lowest	2059 (20)
Mid-low	2233 (22)
Mid	2097 (20)
Mid-high	1994 (19)
Highest	1940 (19)
Immigrant density^a^	
Least	6876 (66)
Mid	2097 (20)
Most	1288 (12)
Charlson comorbidity score, points	
Missing	2118 (20)
0	5220 (50)
1	1638 (16)
2	758 (7)
≥3	630 (6)
Travel time to thoracic center, min	
<15	3380 (34)
16-30	2554 (26)
31-60	1934 (19)
61-90	1222 (12)
91-120	351 (4)
>120	518 (5)
Topography	
Lower third of esophagus	4086 (39)
Cardia NOS	4013 (39)
Cervical esophagus	147 (1)
Middle third of esophagus	1046 (10)
Esophagus NOS	467 (5)
Upper third of esophagus	352 (3)
Thoracic esophagus	172 (2)
Overlapping lesions of esophagus	61 (1)
Abdominal esophagus	20 (<1)
Histology	
Adenocarcinoma	7059 (68)
Squamous cell carcinoma	2260 (22)
Signet ring cell carcinoma	429 (4)
Other	616 (6)
TNM stage^b^	
0	26 (<2)
I/IA	327 (3)
IB	331 (3)
II/IIA	381 (3)
IIB	747 (7)
III/IIIA	904 (9)
IIIB	439 (4)
IIIC	438 (4)
IV	2410 (23)
Unknown/missing	4361 (42)
Pathological tumor stage^c^	
No.	8819
pT0	55
pT1^d^	353
pT1a	446
pT1b	381
pT2	414
pT3	1172
pT4	56
pTX	41
Missing	5901
Pathological nodal stage^c^	
No.	8819
pN0	1023
pN1	537
pN2	347
pN3	238
pNX	413
Missing	6261
Grade^c^	
No.	8819
1 (Well differentiated)	878
2 (Moderately differentiated)	2783
3-4 (Poorly or undifferentiated)	3044
Cannot be determined	65
Missing	2049

Abbreviation: NOS, not otherwise specified.

^a^ Source: (or adapted from) Statistics Canada Postal Code Conversion File and Postal Code Conversion File Plus (June 2017), which are based on data licensed from Canada Post Corporation. The patient’s postal code at diagnosis was used. Postal codes that were unlinked to the 2006 census would produce missing values.

^b^ From Collaborative Staging database supplemented with pathology data as described in the Methods section.

^c^ From synoptic surgical reports only, which includes esophagectomy or endoscopic resections. TX indicates primary tumor cannot be assessed; T0, no evidence of primary tumor; T1, tumor invades lamina propria, muscularis mucosae, or submucosa; T1a, tumor invades lamina propria or muscularis mucosae; T1b, tumor invades submucosa; T2, tumor invades muscularis propria; T3, tumor invades adventitia; T4, tumor invades adjacent structures.

^d^ A total of 295 reports (84%) of stage pT1 were from biopsy synoptic reports (eg, endoscopic resection rather than esophagectomy). NX indicates regional lymph nodes cannot be assessed; N0, no regional lymph node metastasis; N1-3, metastasis in 1-2 (N1), 3-6 (N2), or 7+ (N3) regional lymph nodes.

Most patients (78%) had tumors in the lower third of esophagus, abdominal esophagus, or cardia, which were predominantly adenocarcinomas (eTable 4 in the Supplement). Stage IV tumors were identified in 23% of patients, stage III in 17%, stage II in 10%, and stage 0-I in 6% of patients, but 42% of patients had no staging information available. The majority of these patients likely had stage IV disease, as they received either no treatment (34%) or nonsurgical treatment (44%) (eTable 5 in the Supplement).

### Health Care Use and Wait Times

The time from the earliest health care encounter related to the esophageal cancer workup to the start of treatment (or 2 months after diagnosis if untreated) was a median 91 days (IQR, 55-157 days). Chest radiography was performed in 7618 patients (74%), and in 50%, this was their first assessment. A total of 9661 patients (93%) had a CT scan a median of 27 days (IQR, 7-80 days) after their first encounter (Table 2). Positron emission tomography (PET)/CT was performed in 4635 patients (45%) a median of 63 days (IQR, 36-127 days) into their evaluation (84% stage 0; 72% stage I; 68% stage II; 74% stage III, and 32% for patients with stage IV). A total of 95% of PET/CT scans were performed at 5 hospitals (eTable 6 in the Supplement). Use of endoscopic ultrasonography overall was 12% (performed in 1231 cases) but ranged from 3% (6 of 182 cases) to 83% (190 of 230 cases) depending on the institution (eTable 6 in the Supplement).

**Table 2.** Healthcare Encounters (N = 10 364)

Variable	No. (%)	Time since first encounter, median (IQR), d^a^
Diagnostic tests		
Chest x-ray	7622 (74)	0 (0-53)
Other diagnostic examination	3354 (32)	14 (0-90)
Biopsy^b^	10 114 (98)	26 (3-85)
CT scan (chest, abdomen, pelvis)	9665 (93)	27 (7-80)
PET/CT scan	4635 (45)	63 (36-127)
Endoscopic ultrasonography	1234 (12)	70 (35-134)
Laparoscopy	160 (2)	78 (39-150)
Laryngobronchoscopy	1715 (15)	77 (38-144)
Polypectomy	239 (2)	114 (63-170)
Consultations and visits		
General surgeon	6037 (58)	18 (0-65)
Internist	5940 (57)	21 (0-69)
Gastroenterologist	5034 (49)	21 (0-85)
General thoracic surgeon	5517 (53)	49 (21-113)
Radiation oncologist	7159 (69)	60 (32-120)
Medical oncologist	5135 (50)	61 (33-121)
Treatment^c^	8222 (79)	93 (56-159)

Abbreviations: CT, computed tomography; IQR, interquartile range; PET, positron emission tomography.

^a^ Calculated as the time from the first health care encounter until the event. The first visit was defined as the first health care visit within 6 months before the diagnosis date (inclusive) related to an esophageal cancer workup.

^b^ Biopsy includes endoscopy, esophogastroduodenoscopy, or gastroscopy.

^c^ Includes the first of either esophagectomy, endoscopic mucosal resection, systemic therapy, or radiation.

Initial consultations with a general surgeon (n = 6037, 58%), gastroenterologist (n = 5034, 48%), or internist (n = 5940, 48%) occurred at a median of 2 to 3 weeks (IQR, 0 to 9-12 weeks) after starting the diagnostic workup. Consultation with a general thoracic surgeon (n = 5517, 53%) occurred later (median, 49 days; IQR, 21-113 days), whereas consultation with a medical oncologist (n = 5135, 49%) or radiation oncologist (n = 7159, 69%) occurred after a median of 2 months (IQR, 32-121 months). All 3 oncology specialists were seen by 2778 patients (27%), whereas 1514 (15%) did not consult with any of these specialists. The time from the earliest health care encounter until diagnosis (the diagnostic interval) was a median of 37 days (IQR, 7-108 days) (eTable 7 in the Supplement).

### Treatment

**Figure 1.**
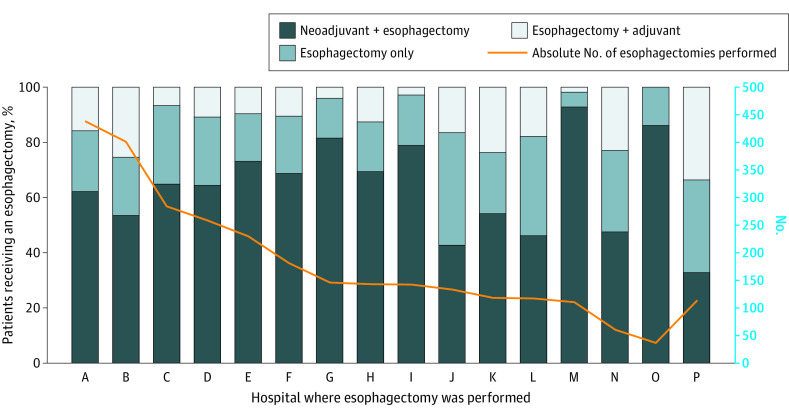
Regional variability in treatment modality Variability in the proportion of patients receiving an esophagectomy alone, esophagectomy following neoadjuvant treatment, or esophagectomy followed by adjuvant treatment (neoadjuvant or adjuvant treatment includes any combination of chemotherapy or radiation). Each bar corresponds to a center where esophagectomies were performed. The far-right bar, P, represents a combination of all hospitals performing more than 30 esophagectomies over the entire study period. The yellow line (number of patients) corresponds to the right y-axis.

A total of 8222 patients (79%) received treatment within 1 year of diagnosis a median of 46 days (IQR, 29-66 days) after diagnosis (Table 3). The most common treatment modalities were radiation alone (n = 1995; 19% of patients), chemoradiation (n = 1882; 19%), neoadjuvant chemoradiation followed by surgery (n = 1849; 18%), chemotherapy alone (n = 865; 8%), esophagectomy without adjuvant or neoadjuvant treatment (n = 668; 6%), esophagectomy or EMR followed by adjuvant therapy (n = 488; 5%), and EMR alone (n = 466; 4%). Overall, esophagectomy was performed in 3047 patients (29%), 114 of whom received their esophagectomy after an EMR that occurred a median of 81 days earlier. Whether a patient received esophagectomy alone, esophagectomy after neoadjuvant therapy, or esophagectomy followed by adjuvant therapy varied by hospital (eg, esophagectomy alone ranged from 5%-39%, neoadjuvant therapy ranged from 33%-93%, and adjuvant therapy ranged from 0%-34%) (*P* < .001 by χ^2^ test) (Figure 1). Patients who received chemoradiation alone (n = 1882) were more likely to have squamous cell carcinoma (n = 578) than those who did not (31% vs 20%) (*P* < .001 by χ^2^ test).

**Table 3.** Treatment Modality

Variable	No. (%) of patients	Time until treatment start, median (IQR), d
Total No.	10 364	NA
Any treatment^a^	8222 (79)	46 (29-66)
Excluding EMR on the diagnosis date	7965 (77)	47 (31-67)
Patients receiving esophagectomy		
Total	3047 (29)	NA
Esophagectomy following EMR	114 (1)	114 (81-171)
EMR was on the diagnosis date	48 (<1)	91 (69-151)
Esophagectomy first (no previous EMR)		
Total	2933 (28)	NA
Esophagogastrectomy only	668 (6)	60 (38-91)
Neoadjuvant therapy	1849 (18)	50 (38-65)
Chemoradiation	1387 (13)	52 (40-66)
Radiation	120 (12)	42 (28-62)
Chemotherapy	342 (3)	49 (35-63)
Adjuvant therapy	416 (4)	55 (34-76)
Chemoradiation	226 (2)	52 (34-74)
Radiation	61 (1)	58 (34-88)
Chemotherapy	129 (1)	55 (36-76)
EMR first		
Total	547 (5)	NA
EMR without chemotherapy or radiation	466 (4)	1 (0-47)
EMR on the diagnosis date	227 (2)	0
EMR after the diagnosis date	239 (2)	43 (29-66)
Neoadjuvant therapy	9 (<1)	41 (22-58)
EMR + adjuvant	72 (1)	13 (0-47)
EMR postdiagnosis + adjuvant	42 (<1)	40 (21-68)
Nonsurgical intervention		
Total	4742 (46)	NA
Chemoradiation	1882 (19)	46 (31-63)
Radiation only	1995 (19)	40 (23-62)
Chemotherapy only	865 (8)	46 (30-66)
No treatment	2142 (21)	NA

Abbreviations: EMR, endoscopic mucosal resection; IQR, interquartile range; NA, not applicable.

^a^ Includes EMR, esophagectomy, radiation, or systemic therapy.

The majority (60%) of patients lived within 30 minutes of a designated thoracic center, 32% lived 30 to 90 minutes away, and 9% lived more than 90 minutes away (eFigure 3 in the Supplement). Travel time was similar for patients who received an esophagectomy compared with those who did not (60% vs 59% resided within 30 minutes; 31% vs 32% resided 30 to 90 minutes; 9% vs 8% resided >90 minutes; *P* = .14). Esophagectomy was performed at a designated thoracic center in 94% of patients (n = 2864) who traveled a median of 29 minutes (IQR, 14-61 minutes; 90th percentile, 105 minutes), whereas patients who received an esophagectomy elsewhere traveled a median of 16 minutes (IQR, 9-29 minutes; 90th percentile, 46 minutes). However, this difference was accounted for by patients residing in remote areas traveling to a designated thoracic center.

Endoscopic mucosal resection was performed in 547 patients (5%), 257 (47%) on the diagnosis date. Most patients had only 1 EMR (n = 306; 56%), 140 (26%) had a second EMR, and 101 (18%) had more than 2 EMRs performed. Most patients who received an EMR (95%) had stage 0-I disease (eTable 5 in the Supplement). Patients who received an EMR without subsequent esophagectomy (n = 433) compared with those who had EMR followed by esophagectomy (n = 114) were older (mean [SD] age, 68 [10.8] years vs 64 [10.2] years) and had more comorbidities (22% vs 11% had more than 2 comorbidities), but there were no differences by sex (81% vs 82% were men), histology (89% vs 95% had adenocarcinoma), urban residence (85% vs 78%), neighborhood income quintile, or neighborhood immigrant density. There was significant regional variability in the use of EMR, with the majority of procedures (n = 377; 69%) being conducted at the one institution that is the major referral center for interventional endoscopy in the province.

In patients with stage 0-III disease, esophagectomy was performed in 2336 patients (65%). Thirty percent of patients with stage II-III disease had nonsurgical treatment. Of the 1726 patients who had no treatment, 168 (5%) were known to have stage 0-III disease.

### Overall Survival

The median patient survival was 10.8 months (95% CI, 10.4-11.1 months). Lower stage was associated with better overall survival, but the median survival for patients with stage I disease was only 53 months, compared with 25 months for stage II, 17 months for stage III, 6 months for stage IV, and 8 months for those with unknown stage (log-rank *P* < .001) (eFigure 4 in the Supplement).

Neoadjuvant therapy followed by esophagectomy had the best overall survival (median survival, 36 months; 95% CI, 32-39 months) (eFigure 4 in the Supplement), whereas median survival for those receiving adjuvant therapy was 27 months (95% CI, 24-30 months; log-rank *P* < .001). The median survival for patients receiving esophagectomy alone was similar to those receiving neoadjuvant therapy (median survival, 36 months; 95% CI, 32-44 months), but there was significant early mortality. The overall survival of patients receiving EMR with and without subsequent esophagectomy was similar (log-rank *P* = .12).

Evaluation of wait times and overall survival revealed that patients starting treatment more than 30 days after diagnosis had better overall survival (median survival, 14.6-17.3 months) than patients treated within 1 month of diagnosis (median survival, 5.0-7.7 months), with the exception of the 414 patients who received treatment at the time of diagnosis (log-rank *P* < .001) (eFigure 4 in the Supplement). Two-thirds of these 414 patients received an EMR as the diagnostic treatment and exhibited excellent survival (median survival was not reached), whereas those who were diagnosed at the time of esophagectomy had an overall survival curve similar to that observed for patients receiving esophagectomy with or without adjuvant treatment, suggesting the date of diagnosis was earlier than that documented by the OCR. Similarly, patients who had a time from diagnosis until treatment of more than 60 days had better overall survival.

**Figure 2.**
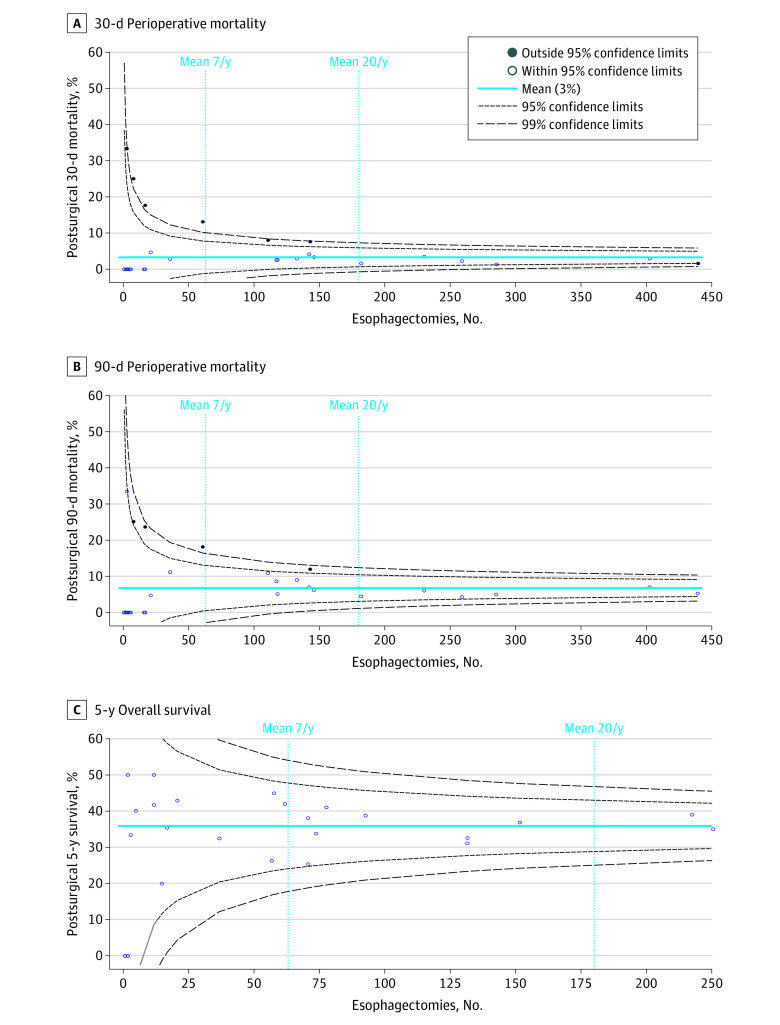
Perioperative mortality and overall survival Funnel plot by hospital performing the patients’ primary esophagectomy. 30-day (A) and 90-day (B) perioperative mortality and 5-year overall survival (C). Estimates of 5-year mortality were restricted to patients diagnosed from January 1, 2010, through December 31, 2014. Vertical reference lines correspond to the mean target number of esophagectomies per year performed for a designated level 1 (20/y) and level 2 (7/y) thoracic cancer center.

Across the province, perioperative mortality after esophagectomy was 3% within 30 days and 7% within 90 days. Perioperative mortality at 30 days was higher for patients receiving esophagectomy at low-volume centers (odds ratio [OR], 3.66, 95% CI, 2.01-6.66) and medium-volume centers (OR, 2.07; 95% CI, 1.33-3.23) compared with high-volume centers (*P* < .001). Similar results were obtained for 90-day mortality (OR, 2.16; 95% CI, 1.34-3.48 for low-volume centers and OR, 1.58; 95% CI, 1.16-2.16 for medium-volume centers compared with high-volume centres; *P* < .001).(Figure 2A and B). In terms of long-term survival, 47% and 34% of patients, respectively, were alive within 3 years and 5 years following esophagectomy, with little regional variability (Figure 2C).

## Discussion

In this cohort study, despite regionalization of esophageal cancer surgery, we found significant regional variability in health care use during the diagnostic workup and in the choice of treatment.

Patients with esophageal cancer waited 1 week longer than patients with lung cancer in Ontario to receive a PET/CT scan and have a consultation with a radiation or medical oncologist, which may have translated into a longer time until treatment (median of 46 vs 40 days).^14^ The reasons for these differences are unknown, despite treatment at the same designated thoracic surgery centers. However, there is limited evidence that longer wait times are associated with worse survival.^15,16,17,18,19^ The association of having a longer time until treatment with better overall survival has been demonstrated previously in patients with cancer and is likely due to a triaging effect.^14,20^ Patients with more overt symptoms are more likely to have advanced disease and be diagnosed or treated urgently, which may only be partially accounted for by stage and is likely to confound any associations between wait times and survival.

The use of EMR may still be underused among patients with stage I disease. There is evidence that EMR is safe and effective in appropriately selected patients, but interpretation from comparative studies using administrative data are limited owing to data quality and unresolved confounding by indication.^21,22^ From administrative data alone, it may not be possible to distinguish a staging EMR followed by planned esophagectomy from a therapeutic EMR followed by salvage esophagectomy.^23^ National Comprehensive Cancer Network guidelines from 2014 support the use of EMR but also recommend antecedent endoscopic ultrasound staging.^24^ Recent clinical practice guidelines from Korea and the European Society for Medical Oncology recommend endoscopic submucosal dissection (ESD) instead of EMR for clinical T1 disease.^25,26^ Evidence suggests that ESD is effective for larger superficial tumors and has a lower rate of recurrence than EMR,^27,28^ but availability of EMR and ESD is limited in Ontario.

For patients eligible to receive esophagectomy, 2 recent systematic reviews^29,30^ have concluded that neoadjuvant chemoradiation is associated with better outcomes compared with adjuvant therapy, including lower recurrence rates and higher overall survival despite greater perioperative mortality. Although our results support these findings, the survival curves are nonproportional and suggest significant confounding remains. The early mortality we observed for patients receiving esophagectomy alone may reflect a subpopulation of patients who were selected for surgery as their primary treatment but were unable to receive neoadjuvant therapy for reasons not discernable from administrative data. Conversely, primary surgery may be suitable for patients with early stage disease and who, therefore, are expected to have better overall survival. Conversely, it may not be possible to distinguish patients who received nonsurgical management alone because their disease was unresectable at diagnosis from patients whose disease progressed during the course of neoadjuvant therapy.^31^ Without more reliable and complete staging information, it is difficult to understand these survival differences. Despite limited evidence to support it, adjuvant therapy was used in 416 patients.^29,31^ Treatment in the real world is not always consistent with the best available evidence (eg, randomized clinical trials), but regional variation analysis may be instrumental to delving into this situation further as a means for potential quality improvement.

Ontario’s thoracic cancer surgery centers are categorized as level 1 or 2 based on the minimum number of esophagectomies (20 per year for level 1 and 7 per year for level 2) in addition to other requirements and standards.^32^ The goal of regionalization was to improve patient outcomes by leveraging the volume-outcome relationship observed with uncommon surgical procedures, such as esophagectomy, while balancing barriers to treatment due to increased travel requirements.^33,34^ We found no evidence that existing travel requirements are a barrier to esophagectomy at a population level. The low volumes at some designated centers may negate the expected benefit of leveraging the potential volume-outcome relationship, but reducing the number of designated centers may conversely leave some patients not willing to travel at all, so careful balance is needed.^35^ Our findings support the volume-outcome relationship for 30-day and 90-day perioperative mortality, but not long-term survival, suggesting that on average, high-volume centers may offer better management of perioperative complications.^36^

Despite regionalization, not all centers have adopted standard-of-care therapy, so from a system perspective, development and use of quality metrics tied to funding may be an effective lever. However, survival for esophageal cancer remains rather dismal, and although there is room for improvement with the use of endoscopic ultrasonography for staging and endoscopic surgery among those eligible to receive it, such changes are unlikely to substantially improve the long-term survival of patients diagnosed with esophageal cancer. More research is needed to identify patients with esophageal cancer before disease progresses to stage IV (the majority of patients). Significant attention has been placed on esophageal cancer screening, but this has not yet been deemed cost-effective due to undersensitive indicators.^37,38^ Other less costly screening modalities are available, but uptake is expected to be low because they are uncomfortable (eg, Cytosponge [Medtronic], an instrument for collecting cells from the esophageal lining), and breath testing for volatile organic compounds is still under development.^39^ Identification of serum biomarkers may offer one minimally invasive option, but more research is needed. Conversely, effective management of chronic gastroesophageal reflux disorder and prevention or treatment of obesity may reduce risk.^40^ These are modifiable risk factors that could be approached on a systemwide basis.

### Limitations

There were some limitations in the present study. Staging data for esophageal cancer are not subject to the Collaborative Staging system at Ontario Health (reserved for lung, breast, colorectal, prostate, and cervix). For this reason, we relied on staging information provided to Ontario Health from the regional cancer centers, and the quality has not been validated against medical records. Regional cancer centers are mandated to provide the stage for any newly diagnosed cancer cases (as opposed to recurrence or progression), but during the study period, did not always do so. Patients who did not attend a regional cancer center during the diagnostic or first-course treatment phase will not have a stage at diagnosis available because there is no arrangement by other facilities to supply staging. We therefore supplemented staging with pathology data for patients who received surgery and for whom an electronic pathology report was available. More accurate and timely staging data are needed if increasing access to endoscopic procedures (EMR or ESD) is important for quality improvement. Our findings may not generalize to jurisdictions where endoscopic diagnostic or therapeutic procedures for esophageal cancer are more commonplace. Another limitation of the present work is that, without information on important confounders (eg, frailty, performance status, patient choice, clinical staging, etc), we were unable to ascertain whether neoadjuvant treatment to esophagectomy vs esophagectomy alone is the preferred treatment modality or whether EMR is as effective as esophagectomy in the real world without undue residual confounding by indication.

## Conclusions

In conclusion, the results of this cohort study suggest that there is significant variation in the diagnosis and management of esophageal cancer across the province of Ontario, despite regionalization of thoracic surgery to sites with regional cancer centers. Variability in care may be associated with resource availability, knowledge, or practice patterns, and further investigation is warranted. Although wait times for diagnosis and treatment appear long, longer wait times were associated with better outcomes. More data are needed to understand whether this association can be explained by appropriate triaging or additional time needed to conduct more extensive investigations in patients without obvious metastatic disease. Endoscopic procedures may improve quality of life by preserving the esophagus, but further study is required to determine whether access to endoscopic therapy affects use rates.

## Notes

Supplement. **eFigure 1.** Cohort Selection **eFigure 2.** Incidence of Esophageal Cancer by Year **eFigure 3.** Patient Travel for Esophagectomy at a Designated Thoracic Center **eFigure 4.** Select Kaplan-Meier Plots for Overall Survival **eTable 1.** Histological Groupings **eTable 2.** Administrative Codes for Health Care Use **eTable 3.** Administrative Codes for Surgery **eTable 4.** Cancer Histology and Location **eTable 5.** Treatment Modality by Stage **eTable 6.** Use of Diagnostic Imaging Among Patients Receiving Esophagectomy **eTable 7.** Health Care Encounters During the Diagnostic and Pretreatment Intervals
