# Supplementary material for: Variation in Diagnosis, Treatment, and Outcome of Esophageal Cancer in a Regionalized Care System in Ontario, Canada
Source: JAMA Netw Open. 2021 Sep 21;4(9):e2126090. doi: 10.1001/jamanetworkopen.2021.26090 (PMC8456383; doi:10.1001/jamanetworkopen.2021.26090)
Supplement: Supplement. — eFigure 1. Cohort Selection eFigure 2. Incidence of Esophageal Cancer by Year eFigure 3. Patient Travel for Esophagectomy at a Designated Thoracic Center eFigure 4. Select Kaplan-Meier Plots for Overall Survival eTable 1. Histological Groupings eTable 2. Administrative Codes for Health Care Use eTable 3. Administrative Codes for Surgery eTable 4. Cancer Histology and Location eTable 5. Treatment Modality by Stage eTable 6. Use of Diagnostic Imaging Among Patients Receiving Esophagectomy eTable 7. Health Care Encounters During the Diagnostic and Pretreatment Intervals [file jamanetwopen-e2126090-s001.pdf]

Supplementary Online Content

Habbous S, Yermakhanova O, Forster K, Holloway CMB, Darling G. Variation in diagnosis, treatment, and outcome of esophageal cancer in a regionalized care system in Ontario, Canada. *JAMA Netw Open*. 2021;4(9):e2126090. doi:10.1001/jamanetworkopen.2021.26090

eFigure 1. Cohort Selection

eFigure 2. Incidence of Esophageal Cancer by Year

eFigure 3. Patient Travel for Esophagectomy at a Designated Thoracic Center

eFigure 4. Select Kaplan-Meier Plots for Overall Survival

eTable 1. Histological Groupings

eTable 2. Administrative Codes for Health Care Use

eTable 3. Administrative Codes for Surgery

eTable 4. Cancer Histology and Location

eTable 5. Treatment Modality by Stage

eTable 6. Use of Diagnostic Imaging Among Patients Receiving Esophagectomy

eTable 7. Health Care Encounters During the Diagnostic and Pretreatment Intervals

This supplementary material has been provided by the authors to give readers additional information about their work.

© 2021 Habbous S et al. *JAMA Network Open.*

eFigure 1. Cohort Selection

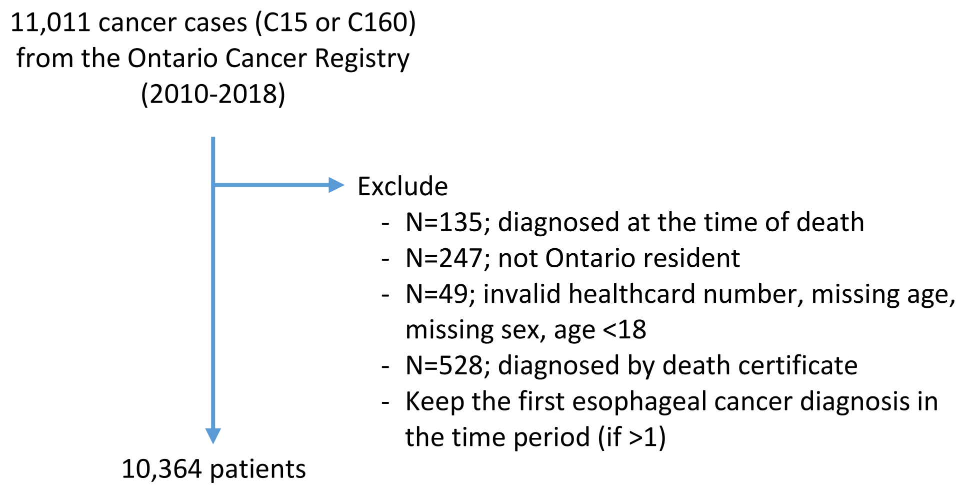

© 2021 Habbous S et al. *JAMA Network Open.*

eFigure 2. Incidence of Esophageal Cancer by Year

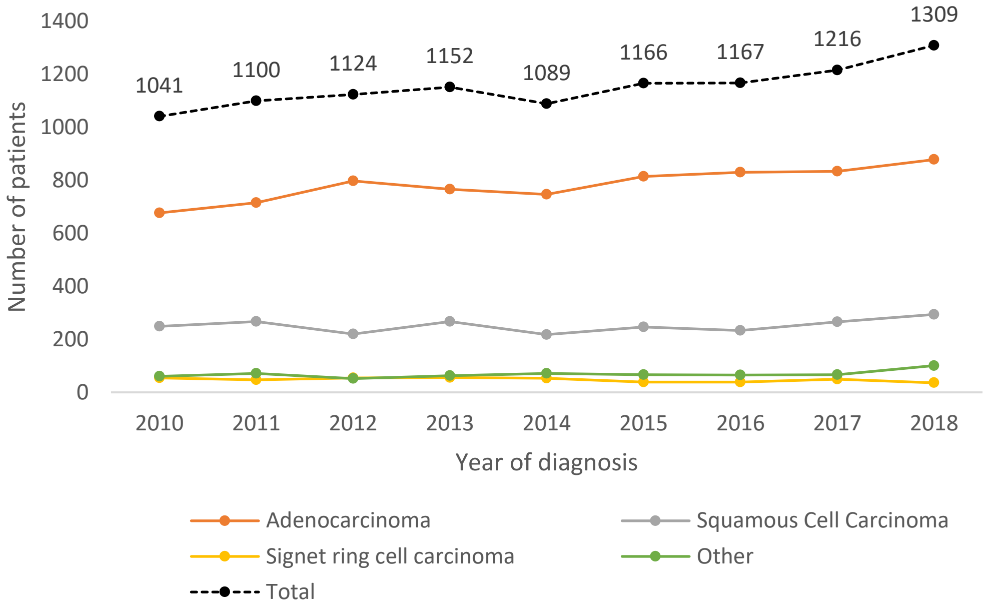

© 2021 Habbous S et al. *JAMA Network Open.*

eFigure 3. Patient Travel for Esophagectomy at a Designated Thoracic Center

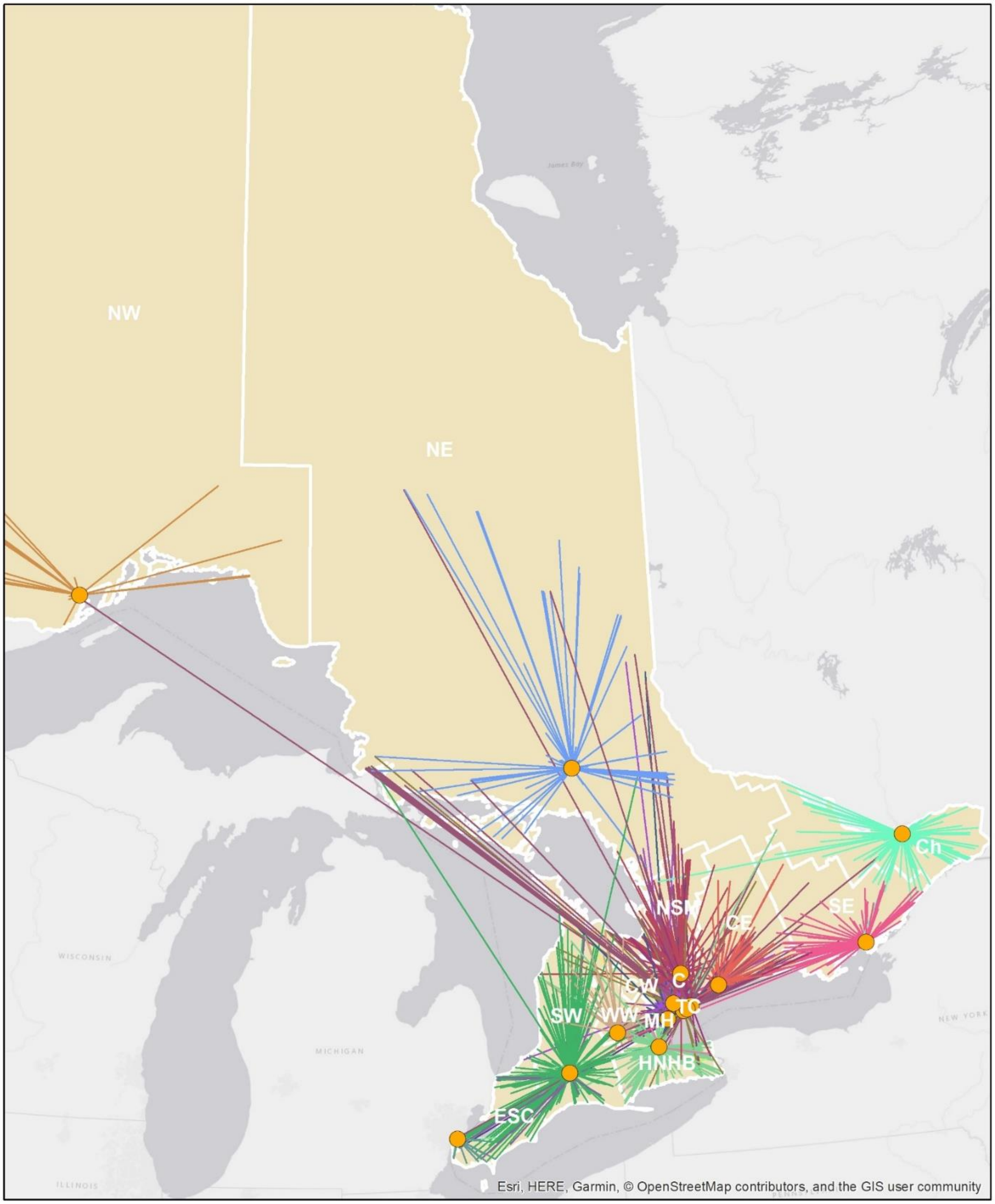

© 2021 Habbous S et al. *JAMA Network Open.*

eFigure 4 Select Kaplan-Meier Plots for Overall Survival

A) by overall stage

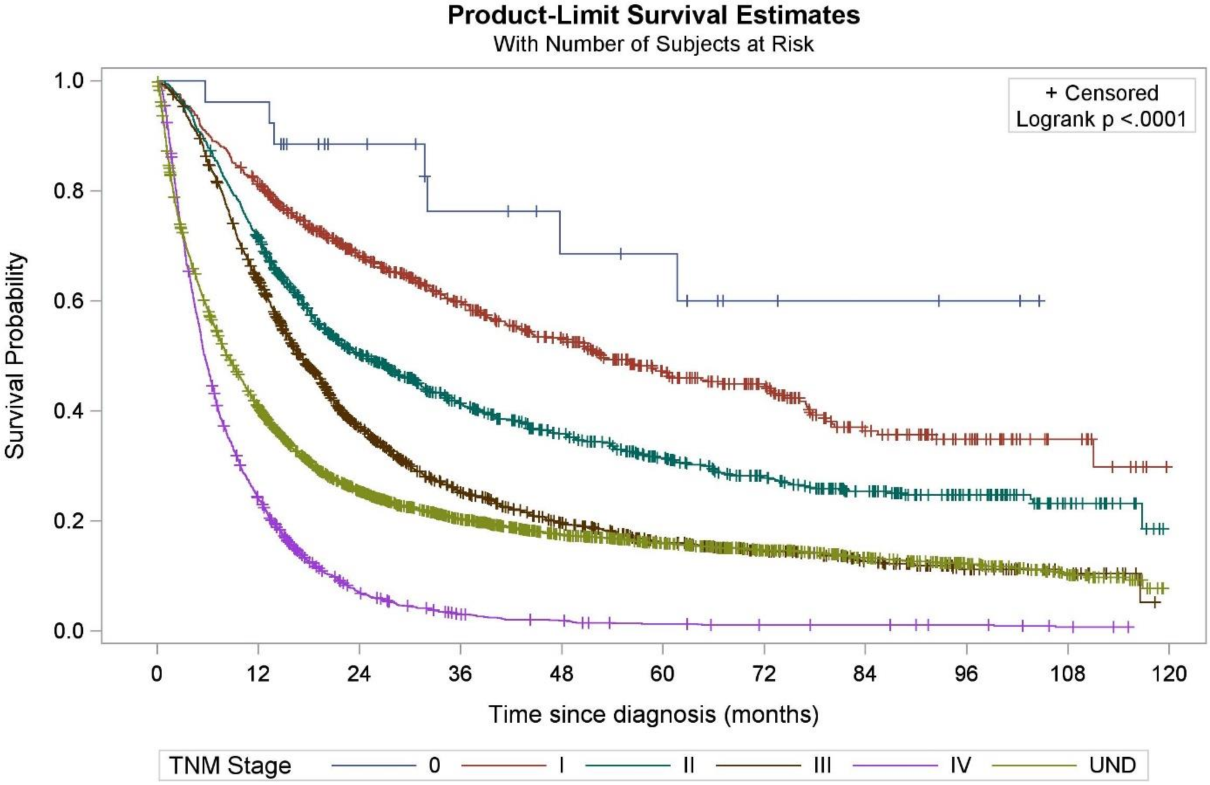

0	26	25	17	12	9	8	4	3	2	0	
I	658	531	375	283	217	150	107	55	31	11	0
II	1127	801	504	351	259	189	136	91	58	21	0
III	1779	1119	572	345	236	155	106	68	32	14	0
IV	2409	569	145	55	31	18	13	11	8	3	0
UND	4356	1744	941	639	456	334	238	157	97	41	0

© 2021 Habbous S et al. *JAMA Network Open.*

B) by overall treatment modality

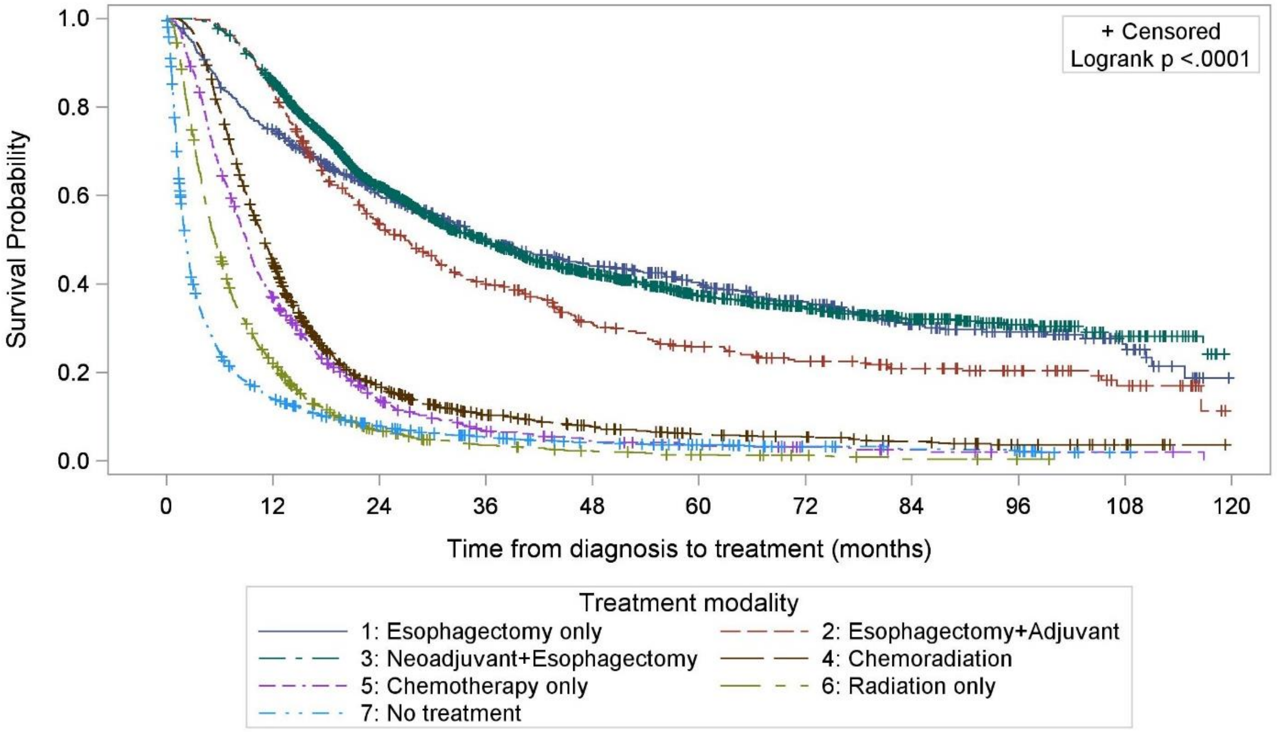

1	668	495	358	269	214	167	120	78	55	21	0
2	416	349	209	146	100	76	59	45	30	14	0
3	1845	1566	961	646	460	314	217	137	76	31	0
4	1882	835	273	140	92	57	43	28	14	7	0
5	864	315	107	45	26	17	11	5	2	2	0
6	1994	431	118	55	32	17	11	3	1	0	
7	2139	293	149	86	59	41	25	17	14	1	0

© 2021 Habbous S et al. *JAMA Network Open.*

C) by receipt of endoscopic mucosal resection (EMR)

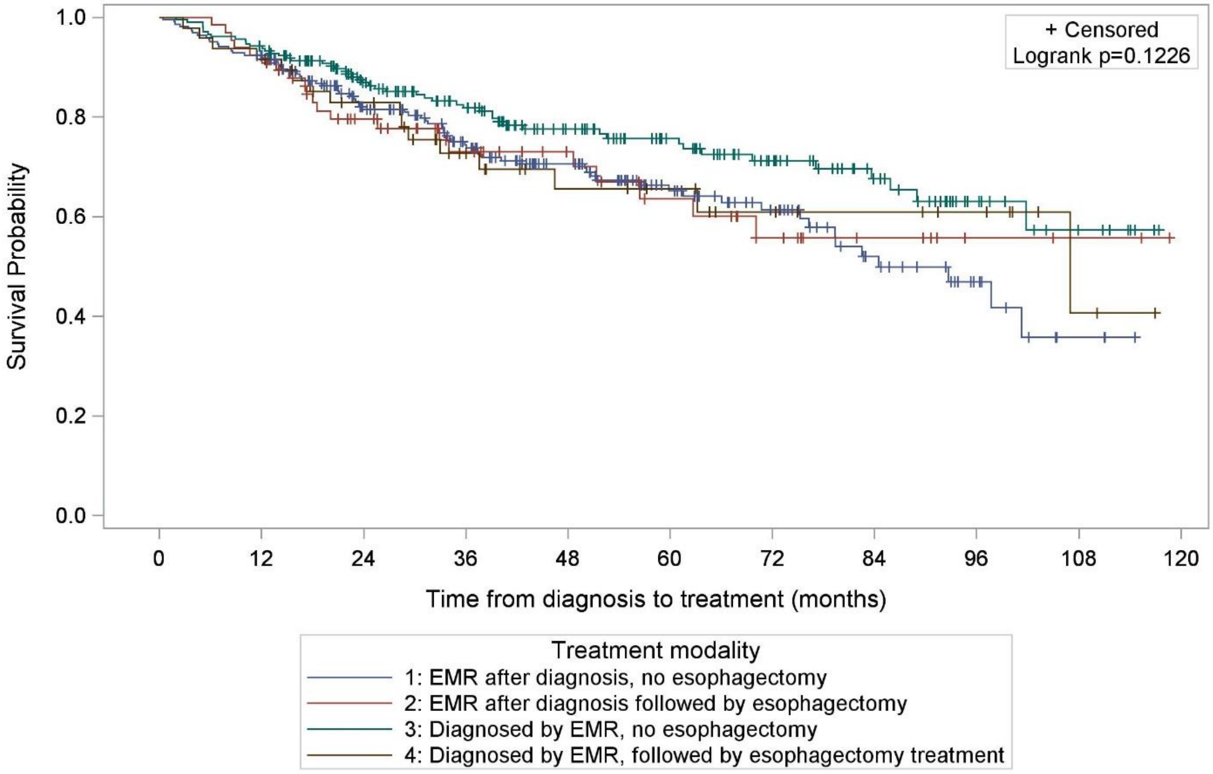

1	224	205	151	120	92	60	42	24	11	3	0
2	66	61	44	31	24	18	12	7	3	2	0
3	209	195	149	123	92	72	53	32	15	7	0
4	48	44	35	24	17	15	11	9	7	2	0

© 2021 Habbous S et al. *JAMA Network Open.*

D) By time from diagnosis until treatment (pre-treatment interval)

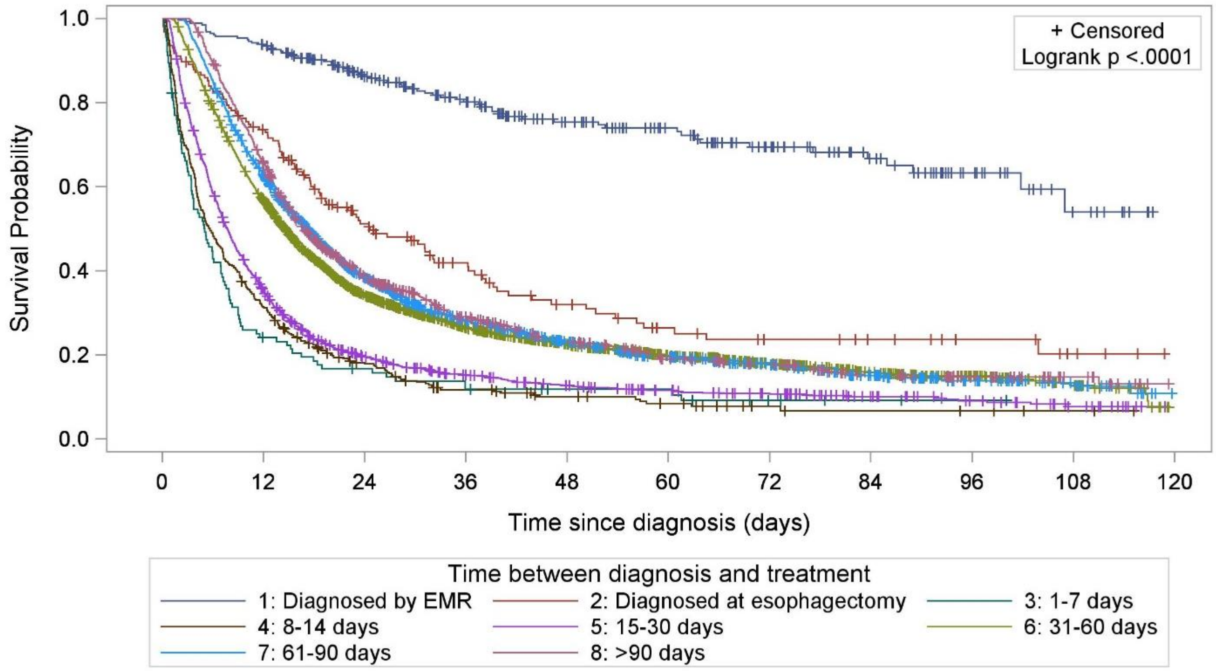

1	258	240	185	148	110	88	65	42	23	9	0
2	156	111	66	44	30	20	15	12	8	4	0
3	113	26	17	12	9	9	4	2	1	0	
4	365	114	56	31	21	15	7	5	4	2	0
5	1351	467	230	156	117	88	69	41	26	12	0
6	3449	1945	1013	677	485	330	225	140	77	30	0
7	1667	1035	551	345	245	172	122	80	45	19	0
8	857	558	287	186	132	91	72	46	30	13	0

© 2021 Habbous S et al. *JAMA Network Open.*

E) by the time from first visit until diagnosis (diagnostic interval)

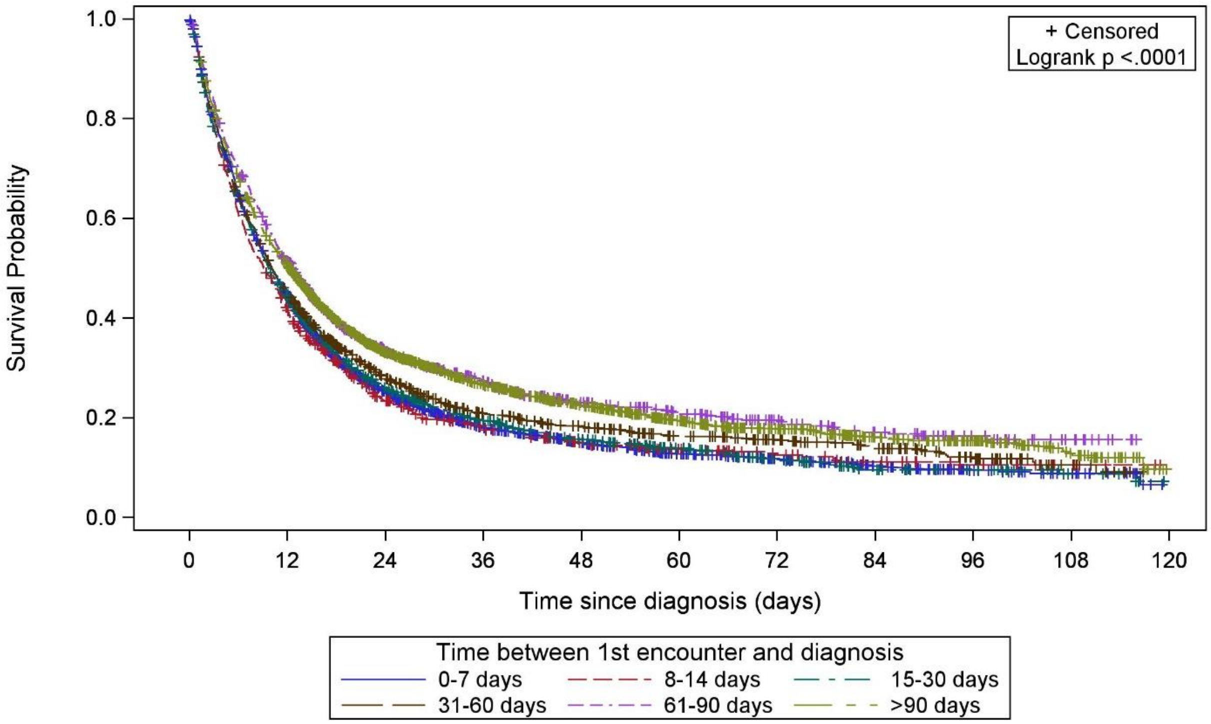

0-7 days	2829	1242	620	370	256	174	122	75	37	18	0
8-14 days	825	338	165	111	82	58	40	27	18	7	0
15-30 days	1272	552	289	186	134	94	61	35	24	8	0
31-60 days	1333	595	330	206	164	120	91	63	35	11	0
61-90 days	1048	531	292	210	144	110	77	49	25	11	0
>90 days	3048	1531	858	602	428	298	213	136	89	35	0

© 2021 Habbous S et al. *JAMA Network Open.*

F) by histological grouping

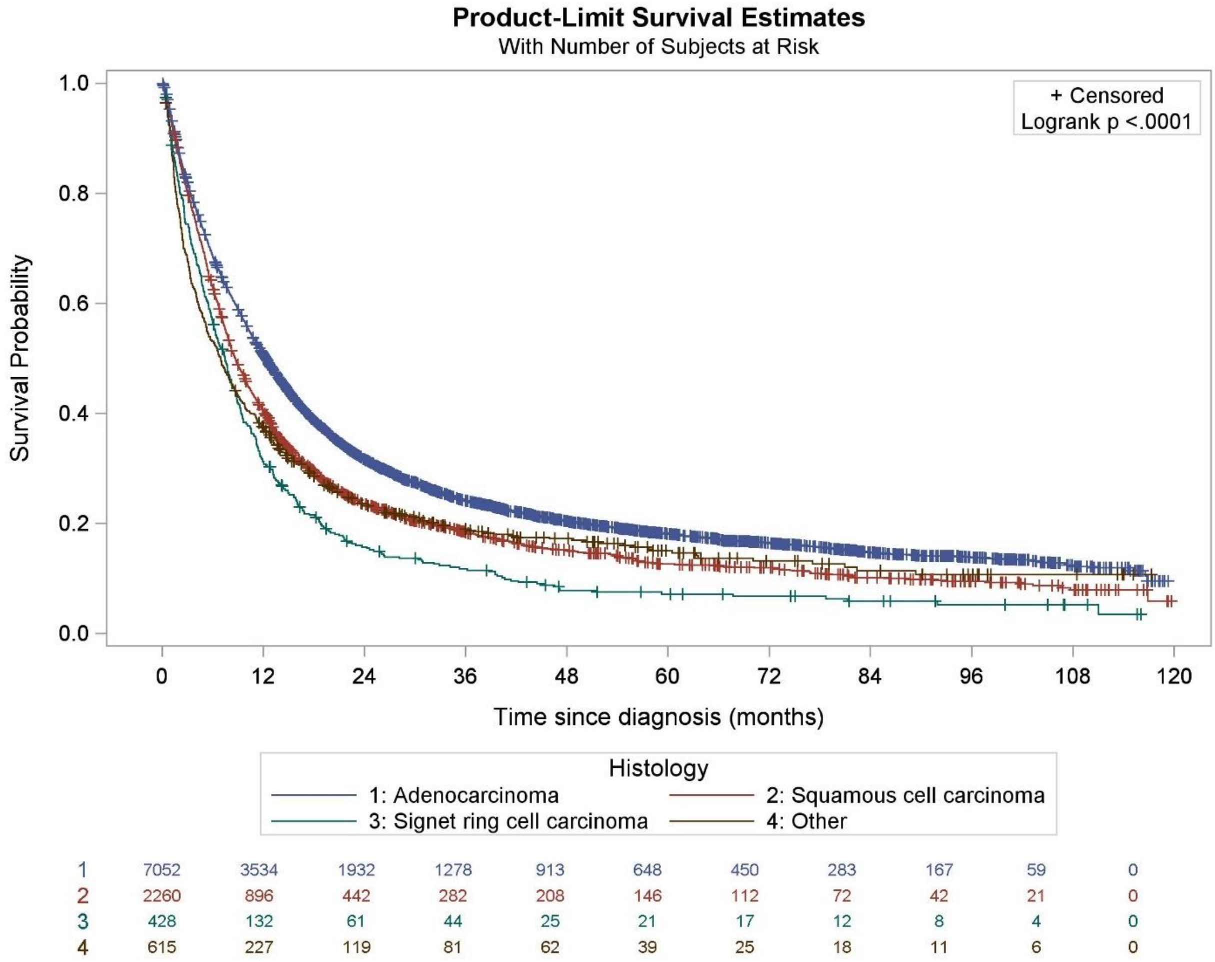

© 2021 Habbous S et al. *JAMA Network Open.*

G) by Charlson comorbidity score

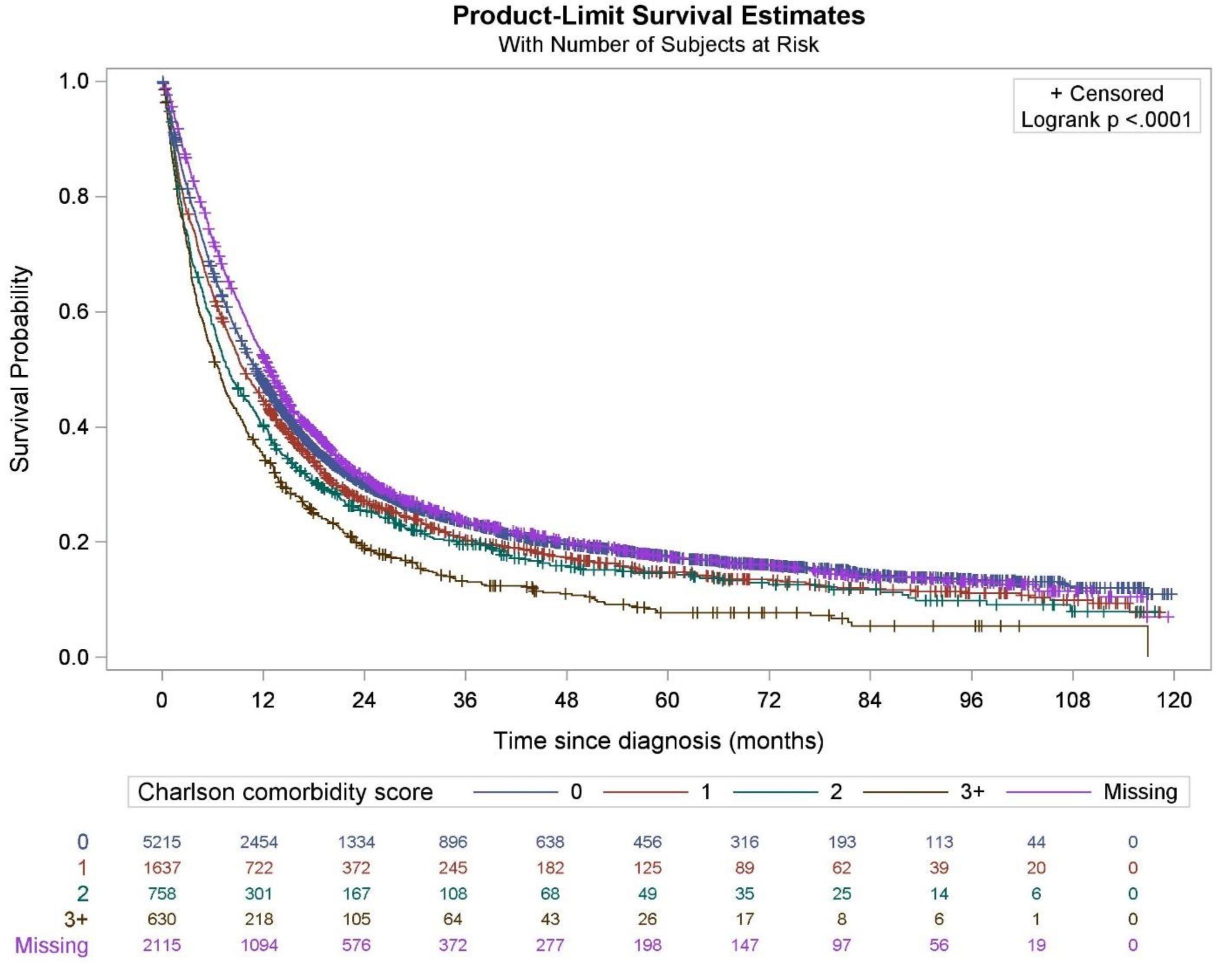

© 2021 Habbous S et al. *JAMA Network Open.*

eTable 1. Histological Groupings

ICD-O-3 code	Description	Type
81403	Adenocarcinoma, NOS	Adenocarcinoma
84803	Mucinous adenocarcinoma	Adenocarcinoma
82553	Adenocarcinoma with mixed subtypes	Adenocarcinoma
81443	Adenocarcinoma, intestinal type	Adenocarcinoma
85743	Adenocarcinoma with neuroendocrine differentiation	Adenocarcinoma
84813	Mucin-producing adenocarcinoma	Adenocarcinoma
80703	Squamous cell carcinoma, NOS	Squamous cell carcinoma
80713	Squamous cell carcinoma, keratinizing, NOS	Squamous cell carcinoma
80723	Squamous cell carcinoma, large cell, nonkeratinizing, NOS	Squamous cell carcinoma
80833	Basaloid squamous cell carcinoma	Squamous cell carcinoma
80743	Squamous cell carcinoma, spindle cell	Squamous cell carcinoma
84903	Signet ring cell carcinoma	Signet ring cell
80003	Neoplasm, malignant	Other
80103	Carcinoma, NOS	Other
81453	Carcinoma, diffuse type	Other
85603	Adenosquamous carcinoma	Other
80203	Carcinoma, undifferentiated, NOS	Other
81483	Glandular intraepithelial neoplasia, grade III – malignant	Other
80773	Squamous intraepithelial neoplasia, low grade – malignant	Other

© 2021 Habbous S et al. *JAMA Network Open.*

eTable 2. Administrative Codes for Health Care Use

**Consultations**		
Gastroenterology	A411, A413, A414, A415, A416, A418, A545, C411, C413, C414, C415, C416, C545, **or**<br>A765, A935, C765, C935, W765 and physician specialty code=33	OHIP
General surgery	A033, A034, A035, A036, C033, C034, C035, C036, W035, W036, **or** A765, A935, C765, C935, W765 and physician specialty code=03	OHIP
General thoracic surgery	A643, A644, A645, A646, C643, C644, C645, C646, W645, W646, **or** A765, A935, C765, C935, W765 and physician specialty code=64	OHIP
Internal medicine	A130, A131, A133, A134, A135, A136, A138, A435, C130, C131, C133, C135, C136, C435, W130, W235, W236, W435	OHIP
Medical oncology	A441, A443, A444, A445, A446, A448, A845, C441, C443, C444, C445, C446, C845, W445, W446, W845, W842, W844, W847, **or** A765, A935, C765, C935, W765 and physician specialty code=44	OHIP
Radiation oncology	A340, A341, A343, A345, A346, A348, A745, C341, C343, C344, C345, C346, C745, **or**<br>A765, A935, C765, C935, W765 and physician specialty code=34	OHIP
**Diagnostic tests**		
Abdominal CT	3OT20, X126, X409, X410	CIHI, OHIP
Biopsy[a]	2NA71, 2NF71, Z399, Z400, Z515, Z327, Z527, Z547, Z548	CIHI, OHIP
Chest CT	3GY20, X125, X406, X407	CIHI, OHIP
Chest x-ray	3GY10, 3OT10, X090, X091, X092, X100, X101	CIHI, OHIP
Other diagnostic exam	X103, X104, X105, X106, X107, X108, X109, X110	OHIP
Esophagogastroduodenoscopy alone (no biopsy)*	Z399, Z400 and no **E674, E675, E702, E703 or E799** billed on the same day	OHIP
Esophagoscopy (no biopsy) *	Z515 and no **E702 or E703, or E799** billed on the same date	OHIP
Gastroscopy (no biopsy)*	Z547, Z528, Z527 and no **E674, or E675** billed on the same date	OHIP
Laryngobronchoscopy (no biopsy)**	Z327 if no E638 billed on the same date	OHIP
PET/CT	J710 and all records from PET registry	OHIP, PET Registry
Pelvic CT	X231, X232, X233	OHIP
Polypectomy*	Z399, Z400 and E703 or E799 billed on the same day<br>Z515 and E703 billed on the same date<br>Z527 and E674 or E675 billed on the same date	OIHP
Endoscopic ultrasound	S236	OHIP

[a] includes biopsies from any approach (e.g. endoscopy, esophagogastroduodenoscopy, or gastroscopy)
*If esophagogastroduodenoscopy only/endoscopy only/gastroscopy only **and** polypectomy/biopsy is billed on the same day - the polypectomy or biopsy is chosen. If biopsy and polypectomy is billed on the same day - the biopsy is chosen.
** If esophagogastroduodenoscopy only/endoscopy only/gastroscopy only/polypectomy/biopsy **and** laryngobrochoscopy were billed on the same day both are counted.

© 2021 Habbous S et al. *JAMA Network Open.*

eTable 3. Administrative Codes for Surgery

**Endoscopic resection (DAD or NACRS)**	
1NA87BA	Endoscopic resection
**Esophagectomy or gastrectomy (OHIP)**	
S090	Oesophagus- excision-total thoracic oesophageal resection
S089	Oesophagus- excision.part.resect.&reconst.incl.intest.transplant
S123	Stomach- excision -gastrectomy-part/subtotal-distal
S125	Stomach- excision -gastrectomy-part/subtotal-proximal
S129	Stomach- excision -conversion of previous gastrectomy to rouz-en-y
**Esophagectomy (DAD or NACRS)**	
1NA87DB	Excision partial, esophagus with anastomosis using endoscopic abdominal approach [e.g. open cervical with laparoscopic approach]
1NA87DBXXF	Excision partial, esophagus with interpositional (intestine) flap using endoscopic abdominal approach [e.g. open cervical with laparoscopic approach]
1NA87DBXXG	Excision partial, esophagus with gastric pull up using endoscopic abdominal approach [e.g. open cervical with laparoscopic approach]
1NA87EZ	Excision partial, esophagus with anastomosis using endoscopic thoracic approach [e.g. open cervical with thoracoscopic approach]
1NA87FA	Excision partial, esophagus with anastomosis using combined endoscopic thoracoabdominal approach
1NA87FAXXF	Excision partial, esophagus with interpositional (intestine) flap using combined endoscopic thoracoabdominal approach
1NA87FAXXG	Excision partial, esophagus with gastric pull up using combined endoscopic thoracoabdominal approach
1NA87LB	Excision partial, esophagus with anastomosis using open cervical with abdominal [transhiatal] approach
1NA87LBXXF	Excision partial, esophagus with interpositional (intestine) flap using open cervical with abdominal [transhiatal] approach
1NA87LBXXG	Excision partial, esophagus with gastric pull up using open cervical with abdominal [transhiatal] approach
1NA87LP	Excision partial, esophagus with anastomosis using open cervical approach
1NA87QB	Excision partial, esophagus with anastomosis using open thoracic approach [e.g. cervicothoracic approach]
1NA87QBXXF	Excision partial, esophagus with interpositional (intestine) flap using open thoracic approach [e.g. cervicothoracic approach]
1NA87QBXXG	Excision partial, esophagus with gastric pull up using open thoracic approach [e.g. cervicothoracic approach]
1NA87QF	Excision partial, esophagus with anastomosis using combined open (cervico)thoracoabdominal approach
1NA87QFXXF	Excision partial, esophagus with interpositional (intestine) flap using combined open (cervico)thoracoabdominal approach
1NA87QFXXG	Excision partial, esophagus with gastric pull up using combined open (cervico)thoracoabdominal approach
1NA89DB	Excision total, esophagus using endoscopic abdominal approach with anastomosis
1NA89DBXXF	Excision total, esophagus using endoscopic abdominal approach [e.g. open cervical with laparoscopic approach] with interpositional (intestine) flap

© 2021 Habbous S et al. *JAMA Network Open.*

1NA89DBXXG	Excision total, esophagus using endoscopic abdominal approach [e.g. open cervical with laparoscopic approach] with gastric pull up
1NA89FA	Excision total, esophagus using combined endoscopic thoracoabdominal approach with anastomosis
1NA89FAXXF	Excision total, esophagus using combined endoscopic thoracoabdominal approach with interpositional (intestine) flap
1NA89FAXXG	Excision total, esophagus using combined endoscopic thoracoabdominal approach with gastric pull up
1NA89LB	Excision total, esophagus using open cervical with abdominal [transhiatal] approach with anastomosis
1NA89LBXXF	Excision total, esophagus using open cervical with abdominal [transhiatal] approach with interpositional (intestine) flap
1NA89LBXXG	Excision total, esophagus using open cervical with abdominal [transhiatal] approach with gastric pull up
1NA89QF	Excision total, esophagus using combined open (cervico)thoracoabdominal approach with anastomosis
1NA89QFXXF	Excision total, esophagus using combined open (cervico)thoracoabdominal approach with interpositional (intestine) flap
1NA89QFXXG	Excision total, esophagus using combined open (cervico)thoracoabdominal approach with gastric pull up
1NA91DB	Excision radical, esophagus using endoscopic abdominal approach [e.g. open cervical with laparoscopic approach] with anastomosis
1NA91DBXXF	Excision radical, esophagus using endoscopic abdominal approach [e.g. open cervical with laparoscopic approach] with interpositional (intestine) flap
1NA91DBXXG	Excision radical, esophagus using endoscopic abdominal approach [e.g. open cervical with laparoscopic approach] with gastric pull up
1NA91FA	Excision radical, esophagus using combined endoscopic thoracoabdominal approach with anastomosis
1NA91FAXXF	Excision radical, esophagus using combined endoscopic thoracoabdominal approach with interpositional (intestine) flap
1NA91FAXXG	Excision radical, esophagus using combined endoscopic thoracoabdominal approach with gastric pull up
1NA91LB	Excision radical, esophagus using open cervical with abdominal [transhiatal] approach with anastomosis
1NA91LBXXF	Excision radical, esophagus using open cervical with abdominal [transhiatal] approach with interpositional (intestine) flap
1NA91LBXXG	Excision radical, esophagus using open cervical with abdominal [transhiatal] approach with gastric pull up
1NA91QF	Excision radical, esophagus using combined open (cervico)thoracoabdominal approach with anastomosis
1NA91QFXXF	Excision radical, esophagus using combined open (cervico)thoracoabdominal approach with interpositional (intestine) flap
1NA91QFXXG	Excision radical, esophagus using combined open (cervico)thoracoabdominal approach with gastric pull up
1NA87DA	Excision partial, esophagus using apposition technique [e.g. suturing] or no closure require using endoscopic [thoracic] approach
1NA87DC	Excision partial, esophagus abdominal [level] anastomosis (esophago-esophagostomy, esophago-gastrostomy) using endoscopic abdominal approach [includes: open cervical with laparoscopic approach]

© 2021 Habbous S et al. *JAMA Network Open.*

1NA87DD	Excision partial, esophagus cervical [level] anastomosis (esophago-esophagostomy) using endoscopic abdominal approach [includes: open cervical with laparoscopic approach]
1NA87EY	Excision partial, esophagus cervical [level] anastomosis (esophago-esophagostomy) using endoscopic thoracic approach [thoracoscopic, or cervical with thoracoscopic]
1NA87FB	Excision partial, esophagus abdominal [level] anastomosis (esophago-esophagostomy, esophago-gastrostomy) using combined endoscopic thoraco-abdominal approach
1NA87FC	Excision partial, esophagus thoracic [level] anastomosis (esophago-esophagostomy) using combined endoscopic thoraco-abdominal approach (may involve mixing both open and endoscopic approaches to thorax and abdomen with or without a cervical incision)
1NA87LD	Excision partial, esophagus abdominal [level] anastomosis (esophago-esophagostomy, esophago-gastrostomy) using open abdominal approach [includes cervical with abdominal approach, transhiatal approach]
1NA87LE	Excision partial, esophagus cervical [level] anastomosis (esophago-esophagostomy) using open abdominal approach [includes cervical with abdominal approach, transhiatal approach]
1NA87QC	Excision partial, esophagus cervical [level] anastomosis (esophago-esophagostomy) using open thoracic approach [includes: open cervicothoracic approach]
1NA87QD	Excision partial, esophagus thoracic [level] anastomosis (esophago-esophagostomy) using open thoracic approach [includes: open cervicothoracic approach]
1NA87QG	Excision partial, esophagus abdominal [level] anastomosis (esophago-esophagostomy, esophago-gastrostomy) using combined open thoraco-abdominal approach [includes: open cervical with thoraco-abdominal approach]
1NA87QH	Excision partial, esophagus thoracic [level] anastomosis (esophago-esophagostomy) using combined open thoraco-abdominal approach [includes: open cervical with thoraco-abdominal approach]
1NA88DCXXG	1NA88DCXX-Excision partial with reconstruction, esophagus endoscopic abdominal approach with interpositional intestinal [free] flap (*)/ with gastric pull-up
1NA88FCXXG	1NA88FCXX - Excision partial with reconstruction, esophagus endoscopic thoracoabdominal approach with interpositional intestinal [free] flap (*)/ with gastric pull-up
1NA88LBXXF	1NA88LBXX - Excision partial with reconstruction, esophagus open cervical with abdominal approach [includes: transhiatal approach] with interpositional intestinal [free] flap (*)/ with gastric pull-up
1NA88LBXXG	1NA88LBXX - Excision partial with reconstruction, esophagus open cervical with abdominal approach [includes: transhiatal approach] with interpositional intestinal [free] flap (*)/ with gastric pull-up
1NA88QFXXF	1NA88QFXX - Excision partial with reconstruction, esophagus open thoraco-abdominal approach [includes: cervical with thoraco-abdominal approach] with interpositional intestinal [free] flap (*)/ with gastric pull-up
1NA88QFXXG	1NA88QFXX - Excision partial with reconstruction, esophagus open thoraco-abdominal approach [includes: cervical with thoraco-abdominal approach] with interpositional intestinal [free] flap (*)/ with gastric pull-up
1NA90LBXXF	1NA90LBXX-Excision total with reconstruction, esophagus open cervical with abdominal approach [includes: transhiatal approach] with interpositional intestinal [free] flap (*)/ with gastric pull-up
1NA90LBXXG	1NA90LBXX-Excision total with reconstruction, esophagus open cervical with abdominal approach [includes: transhiatal approach] with interpositional intestinal [free] flap (*)/ with gastric pull-up
1NA90QFXXF	1NA90QFXX -Excision total with reconstruction, esophagus open thoraco-abdominal approach [includes: cervical with thoraco-abdominal approach] with interpositional intestinal [free] flap (*)/ with gastric pull-up

© 2021 Habbous S et al. *JAMA Network Open.*

1NA90QFXXG	1NA90QFXX -Excision total with reconstruction, esophagus open thoraco-abdominal approach [includes: cervical with thoraco-abdominal approach] with interpositional intestinal [free] flap (*)/ with gastric pull-up
1NA92LBXXG	1NA92LBXX-Excision radical with reconstruction, esophagus open cervical with abdominal approach [Includes: transhiatal] with interpositional intestinal [free] flap (*)/ with gastric pull-up
1NA92QFXXF	1NA92QFXX-Excision radical with reconstruction, esophagus open thoraco-abdominal approach [Includes: cervical with thoraco-abdominal approach] with interpositional intestinal [free] flap (*)/ with gastric pull-up
1NA92QFXXG	1NA92QFXX-Excision radical with reconstruction, esophagus open thoraco-abdominal approach [Includes: cervical with thoraco-abdominal approach] with interpositional intestinal [free] flap (*)/ with gastric pull-up
OHIP – Ontario Health Insurance Program database; DAD – Discharge Abstract Database (inpatient hospital procedures); NACRS – National Ambulatory Care Reporting System (outpatient or same-day-surgery hospital procedures)	

© 2021 Habbous S et al. *JAMA Network Open.*

eTable 4. Cancer Histology and Location

Cancer Type	Cancer Location						
	Upper or middle esophagus		Lower esophagus		Other		Total
Adenocarcinoma	350	5%	6416	91%	293	4%	7059
Signet ring cell carcinoma	7	2%	409	95%	13	3%	429
Squamous cell carcinoma	1294	57%	802	35%	164	7%	2260
Other	66	11%	492	80%	58	9%	616
Total	1717	17%	8119	78%	528	5%	10364

© 2021 Habbous S et al. *JAMA Network Open.*

eTable 5. Treatment Modality by Stage

Stage	All patients	Endoscopic resection[a]	Esophagectomy[a]	Non-surgical treatment[b]	No treatment
I or 0	684 (6%)	58 (8%)	456 (67%)	135 (20%)	35 (5%)
II	1,128 (11%)	12 (1%)	717 (64%)	343 (30%)	56 (5%)
III	1,781 (17%)	8 (1%)	1,163 (65%)	533 (30%)	77 (4%)
IV	2,410 (23%)	9 (<1%)	111 (5%)	1,809 (75%)	481 (20%)
Unknown	4,361 (42%)	460 (11%)	486 (11%)	1,922 (44%)	1,493 (34%)
Total	10,364 (100%)	547 (5%)	2,933 (28%)	4,742 (46%)	2,142 (21%)

Stage 0 combined with stage 1 due to small cells
[a] as the first surgical intervention. This includes neoadjuvant and adjuvant treatment
[b] includes radiation alone, systemic therapy alone, or chemoradiation

© 2021 Habbous S et al. *JAMA Network Open.*

eTable 6. Use of Diagnostic Imaging Among Patients Receiving Esophagectomy

**Location where PET/CT was performed**[a]	**N (%) receiving PET/CT**	
Missing[b]	804 (17%)	
A	845 (18%)	
B	773 (17%)	
C	717 (16%)	
D	583 (15%)	
E	611 (13%)	
Other (e.g. private labs)	83 (2%)	
**Hospital where esophagectomy was performed**[a,c]	**Esophagectomy (N)**	**N (%) receiving endoscopic ultrasound**
A	230	190 (83%)
B	259	129 (50%)
C	285	116 (41%)
D	117	29 (25%)
E	439	107 (24%)
F	146	19 (13%)
G	142	17 (12%)
H	143	11 (8%)
I	133	9 (7%)
J	402	20 (5%)
K	182	6 (3%)
L	61	<6
M	118	<6
N	111	<6
O	36	<6
Hospitals with less than 30 surgeries	*111*	*10 (9%)*

[a] hospitals were de-identified and the identifier in the upper half of the table does not necessarily correspond with the same identifier in the lower half of the table.
[b] all missing locations were associated with a PET/CT captured through administrative billing (OHIP) rather than the PET Registry
[c] location where endoscopic ultrasound was performed was often missing, as this was ascertained from OHIP. The hospital where the esophagectomy was performed was used instead.
PET/CT – positron emission tomography/computed tomography

© 2021 Habbous S et al. *JAMA Network Open.*

eTable 7. Health Care Encounters During the Diagnostic and Pretreatment Intervals

	Diagnostic interval		Pre-treatment interval	
	**All patients (N=10,364)**	**Time until diagnosis starts Median (IQR) days**	**All patients (N=10,364)**	**Time since diagnosis Median (IQR) days**
**Consultations and visits[a]**	**9,372 (90%)**	**11 (0, 76)**		
Gastroenterologist	3,570 (34%)	1 (0, 29)	2,952 (28%)	9 (3, 22)
General surgeon	4,146 (40%)	11 (1, 50)	4,207 (41%)	12 (6,21)
General thoracic surgeon	1,053 (10%)	7 (0, 36)	4,995 (48%)	16 (9, 27)
Internist	3,770 (36%)	17 (0, 94)	4,214 (41%)	17 (6, 34)
Medical oncologist	320 (3%)	41 (8, 118)	4,957 (48%)	28 (17, 42)
Radiation oncologist	523 (5%)	57 (18, 130)	6,908 (67%)	26 (17, 40)
**Diagnostic evaluation[a]**	**10,070 (97%)**	**20 (0, 75)**		
Biopsy[b]	9,162 (88%)	0 (0, 0)	–	–
CT scan (chest, abdomen, pelvis)	4,439 (43%)	8 (0, 42)	7,706 (74%)	9 (4, 20)
Chest x-ray	5,441 (52%)	34 (7, 90)	–	–
Other diagnostic exam	2,483 (24%)	20 (8, 45)	–	–
PET/CT	242 (2%)	29 (10, 49)	4,395 (42%)	29 (20, 40)
Laparoscopy	7 (<1%)	16 (8, 123)	153 (1%)	34 (24, 54)
Laryngobronchoscopy	266 (3%)	0 (0, 27)	1,497 (14%)	33 (17, 56)
Polypectomy	129 (1%)	0 (0, 49)	–	–
Endoscopic ultrasound	192 (2%)	0 (0, 40)	1,049 (10%)	29 (18, 42)
**Any healthcare encounter**	**10,167 (98%)**	**37 (7, 108)**	–	–
**Any healthcare encounter (including the diagnosis date)**	**10,364 (100%)**	**35 (6, 106)**	–	–

CT – computed tomography; PET – positron emission tomography
[a] patients can receive >1 healthcare encounter of the same or different type
[b] Biopsy includes endoscopy, esophagogastroduodenoscopy, or gastroscopy

© 2021 Habbous S et al. *JAMA Network Open.*
